# Coroner's Inquest—Dr. Thomson

**Published:** 1831-01-01

**Authors:** 


### CORONER'S INQUEST.—DR. THOMSON'S RECLAMATION.

"DR. ALEXANDER THOMSON has read with pain the observations of Dr. Johnson, which have evidently been made under a misconception of the evidence delivered by Dr. T. who has this day called to lay before Dr. Johnson the numerous testimonials of approval which he has received from medical men who were present at the inquest on Miss Cashin. Dr. T. trusts Dr. Johnson will publish his answer to the observations. Dr. T. copies a letter which he received from the foreman and jury, on Sept. 2nd.—

"Sir—Allow me in the name of myself and my fellow jury-men, to request your acceptance of our testimony in opposition to the malicious, false and despicable attack made upon you in the London Medical Gazette of last week. We beg you to receive our thanks for your minute and careful examination of the body of the unfortunate Miss C. Cashin, for the patient and clear manner in which you explained to the jury the meaning of every technical term which you employed, and for the unhesitating and open manner in which you answered all questions from whatever person they came, for the deep interest you have taken in this case, and for your whole conduct during the inquest —which we shall not soon forget.

We have the honor, &c."

(Here are the signatures of all the jury and of their foreman.)

---

*Note by the Editor.*—Dr. Thomson has submitted to the perusal of the Editor, a copy of the notes which were taken during the first and the second examination of the body of Miss Cashin, and he has no hesitation in saying that had the substance of these notes been correctly given in the news-papers, it would have saved much altercation and discussion in the medical journals afterwards. The following extract will shew that there was little or no discrepancy of opinion respecting the state of the parts, between Mr. Brodie and Dr. Thomson.

"There was a patch on the back about nine inches long by six broad, irregular at the margin, denuded of cuticle, and of a black colour, intensing towards the centre, and reddening towards the margin. The surface of the sore was hard and dry—the true skin double the natural thickness, indurated and semi-cartilaginous, offering great resistance to the knife. The cellular substance, fasciæ, and muscles were blended into one hard mass, from which they could, with difficulty, be separated by dissection."

Nobody ever doubted Dr. Thomson's education and qualifications, literary as well as professional. We, at least, never doubted them; but we think it is not for the advantage of the public or any of the parties concerned, to go farther into the subject.

